# Short-term variability of biomarkers of proteinase activity in patients with emphysema associated with type Z alpha-1-antitrypsin deficiency

**DOI:** 10.1186/1465-9921-6-47

**Published:** 2005-05-31

**Authors:** Jan Stolk, Barbara Veldhuisen, Laura Annovazzi, Chiara Zanone, Elly M Versteeg, Toine H van Kuppevelt, Willem Nieuwenhuizen, Paolo Iadarola, Maurizio Luisetti

**Affiliations:** 1 Department of Pulmonology, Leiden University Medical Center, Leiden, The Netherlands; 2 Department of Medical Statistics, Leiden University Medical Center, Leiden, The Netherlands; 3 Laboratory of Capillary Electrophoresis, Department of Biochemistry, University of Pavia, Italy; 4 Laboratory of Biochemistry and Genetics, Department of Respiratory Disease, IRCCS San Matteo Hospital, Pavia, Italy; 5 Department of Biochemistry, 194, University Medical Center, NCMLS Nijmegen, The Netherlands; 6 TNO-Prevention and Health, Gaubius Laboratory, Leiden, The Netherlands

**Keywords:** alpha-1-antitrypsin, emphysema, JM403, desmosines, biomarkers

## Abstract

**Background:**

The burden of proteinases from inflammatory cells in the lung of subjects with type Pi ZZ of alpha-1-antitrypsin deficiency is higher than in those without the deficiency. Cross-sectional studies have shown increased levels of biomarkers of extracellular matrix degradation *in vivo*. Longitudinal variability of these biomarkers is unknown but desirable for clinical studies with proteinase inhibitors.

**Methods:**

We measured three different types of biomarkers, including desmosines, elastase-formed fibrinogen fragments and heparan sulfate epitope JM403, in plasma and urine for a period of 7 weeks in a group of 12 patients who participated in a placebo-controlled study to assess the safety of a single inhalation of hyaluronic acid.

**Results:**

Effect of study medication on any of the biomarkers was not seen. Baseline desmosines in plasma and urine correlated with baseline CO diffusion capacity (R = 0.81, p = 0.01 and R = 0.65, p = 0.05). Mean coefficient of variation within patients (CVi) for plasma and urine desmosines was 18.7 to 13.5%, respectively. Change in urinary desmosine levels correlated significantly with change in plasma desmosine levels (R = 0.84, p < 0.01). Mean CVi for fibrinogen fragments in plasma was 20.5% and for JM403 in urine was 27.8%. No correlations were found between fibrinogen fragments or JM403 epitope and desmosines.

**Conclusion:**

We found acceptable variability in our study parameters, indicating the feasibility of their use in an evaluation of biochemical efficacy of alpha-1-antitrypsin augmentation therapy in Pi Z subjects.

## Background

Polymorphonuclear leukocytes (PMNs) play a major role in the pathogenesis of chronic obstructive pulmonary disease (COPD), in particular in emphysema [1]. In subjects with Pi Z type of alpha-1-antitrypsin deficiency (AATD) the burden of PMN and other inflammatory cells in the lung is higher than in those without the deficiency [2,3]. The serum levels of alpha-1-antitrypsin (AAT) found in deficient AAT subjects with phenotypes ranging between Null/Null and MZ correlate with clinical severity of emphysema and suggest that AAT is the most important inhibitor of protease activity in the lung [4]. Proteinases released by inflammatory cells such as PMN and macrophages are able to degrade the extracellular matrix components in lung interstitium, including elastin, proteoglycans and collagens [5]. Although active degradation is difficult to demonstrate *in vivo*, immunohistochemical studies in resected human lung have shown PMN elastase and other proteases present on extracellular matrix components, suggesting that enzyme is in contact with its substrate for degradation [6]. In patients with AATD, such degradation is thought to be more active in the absence of AAT.

The assessment of inflammatory cell-mediated extracellular matrix degradation *in vivo *partly suffers from the lack of specific biochemical markers that reflect proteolysis and thus protease activity *in vivo*. For example, neutrophil elastase can be measured in plasma as antigen concentration or in complex with its inhibitor alpha-1-antitrypsin, but this is only an indication of PMN degranulation and may not be representative of functional extracellular proteolytic activity *in vivo*.

In the past five years, three different concepts of biomarkers of protease activity of extracellular matrix degradation around inflamed alveoli have been published. First, the heparan sulfate specific epitope JM403 was found 10-fold reduced in urine of patients with COPD compared to healthy controls [7]. The decreased urinary content of a specific epitope of heparan sulfate, together with a normal content of heparan sulfate richly present in basement membranes of alveoli suggest a structural alteration or an altered processing of the heparan sulfate molecule in the lungs of patients with emphysema. In view of the biological functions of heparan sulfate, this could lead to destabilisation of the extracellular matrix, facilitating the development of further proteolytic damage to other matrix components [7].

Second, elastin breakdown products were demonstrated in urine and plasma, as a footprint of the degradation of cross-linked elastin [8-10]. Third, large fibrin(ogen) fragments formed by neutrophil elastase-mediated degradation (PMN-FDP) were significantly elevated in plasma of AATD subjects compared to healthy controls, indicating an imbalance in the protease-antiprotease ratio, which allows elastase activity *in vivo *at sites of inflammation where fibrin(ogen) is deposited [11,12].

The aim of the present study was to measure the above three types of biomarkers in a short-term pharmaceutical safety study to assess biomarker variability between and within patients.

## Materials and methods

### Subjects and study design

Twelve patients with Pi ZZ type of AATD participated in a double blind, randomised, placebo-controlled phase I study to investigate the safety and tolerability of a single inhalation of hyaluronic acid (HA), using a Pari Boy compressor and LC nebuliser [13]. Patients were randomised for a single inhalation of a solution of HA (0.003 or 0.01% ETX-100 from CoTherix, Belmont, CA, USA) or placebo. This resulted into 3 blocks of treatment, a block of 4 patients who inhaled 0.003% ETX-100 or placebo, a block of 4 patients who inhaled 0.01% ETX-100 or placebo and another block of 4 patients who inhaled 0.003 or 0.01% ETX-100. These dosages were calculated from dosages that have been used in standard toxicity studies in mice and rats, required by the Food and Drug Administration of the USA, to demonstrate a no effect level in standardized pathological examination. The two inhalations were 15 days apart. The first single inhalation was at baseline visit with overnight stay in the clinic, followed by a control visit on day 9. The second single inhalation was at day 15, followed by another overnight stay in the clinic and by a control visit on day 23. A final visit was scheduled 44 days after the baseline visit. Citrate plasma samples were taken at baseline and days 1, 9, 15, 16, 23 and 44. Urine samples of 24 h period of collection were taken on days 1, 9, 16, 23 and 44.

Patient characteristics are shown in Table 1 (see AdditionalfileRespirResTable1). Although no chest CT's were available, chest X-rays of all patients showed signs of panlobular emphysema and none showed bronchiectasis. All patients were in stable clinical condition in the four weeks preceding the baseline visit and none have ever been treated with AAT replacement therapy.

The study was approved by the Ethical Board of LUMC and all patients gave written informed consent. The study was conducted according to Good Clinical Practice.

### Desmosine assay

The determination of desmosines in urine was performed essentially as previously described [14]. For analysis of these compounds in plasma, the above method was slightly modified. Briefly, plasma samples (1 ml) were deproteinized by addition of 0.45 M trichloroacetic acid (400 μl) and centrifuged at 14,000 rpm for 10 min. Finally, a 100 μl aliquot of each sample was placed into pyrex tubes, evaporated to dryness in vacuo and hydrolyzed by refluxing with 500 μl of twice distilled constant boiling HCl (6 M) at 106°C for 24 h. The hydrolyzed samples were dried under a nitrogen stream, the residue washed four times in de-ionized water and neutralized with 0.5 M Na_2_CO_3_, pH 8.7 to give a final volume of 500 μl. After centrifugation for 15 min at 13,000 rpm, the supernatant was diluted with water (1:1) and labeled for 5 hrs at 45°C in the dark by addition of 50 μl 0.5 mM fluoresceine isothiocyanate (FITC) solution prepared fresh immediately before of use. Samples were then analyzed with capillary electrophoresis and laser-induced fluorescence detection (CE-LIF) using a Beckman P/ACE 2200 (Fullerton, CA, USA) automated system equipped with a LASER MODULE 488 (Beckman) consisting of a 3 mW and a 488 nm air-cooled argon-ion laser. Samples were injected at 3.5 kPa for 10 sec (approximately 10 nl injected) and the separation was carried out at a temperature of 25°C applying a voltage of 30 kV for 25 min. The background electrolyte was 20 mM sodium tetraborate pH 9.0 containing 60 mM sodio dodecyl sulfate and 15% (v/v) methanol. Data were collected and processed using the Beckman System Gold software. The assay is measuring the combination of isodesmosine and desmosine present in both plasma and urine samples. Throughout this manuscript it is referred to as desmosines. The calculated analytical interassay coefficient of variation (CVa) was 4.2 %. Desmosines concentration in urine is normalized to urine creatinine (μg/g creatinine), whereas desmosines concentration in plasma is expressed as μg/L.

### JM403 assay

The heparan sulfate JM403 epitope, defined by monoclonal antibody JM403 was quantified using an inhibition enzyme immunoassay (EIA) as previously described [7]. The EIA was preceded by urine preparation as described [7]. The CVa of the assay was 5 %.

### PMN-FDP assay

Frozen citrate plasma was warmed for 5 – 10 min in a 37°C water bath until the sample was a clear solution. Capture-antibody-coated microtiter plates from Kordia BV, Leiden, The Netherlands were filled with samples diluted in PBS/Tween and incubated for 2 h at room temperature. Bound PMN-FDP was quantified by incubation for 1 h at RT with peroxidase-labelled Mab DD13 in PBS/Tween with 0.1% (w/v) BSA, and the subsequent conversion of TMB/H_2_O_2 _as described [11]. The CVa of the assay was 10%.

### Statistical analysis

After the code of treatment was broken, the change over time from baseline to day 15 and from day 15 to day 44 for each of the treatment groups was calculated for each of the three different biomarkers. To test for a treatment effect of hyaluronic acid on each of the biomarkers, the differences between two time points for a given biomarker in the treatment groups was compared with the differences in the group treated with placebo using an univariate general linear model. A t-test for the inter-subject variability for each of the biomarkers in each of the three treatment blocks showed no statistically significant difference.

Mean values and standard deviations of desmosines, JM 403 and PMN-FDP of all available plasma or urine samples for each of the days of collection were calculated in SPSS version 11. Coefficient of variation within and between patients was calculated.

The correlation between baseline FEV_1_, Kco and baseline value of each of the biomarkers was assessed by the Spearman's rank correlation coefficient.

## Results

Analysis of change over time of any of the three biomarkers for a given treatment with hyaluronic acid did not show a statistically significant difference compared to change by placebo treatment in a univariate general linear statistical model. Therefore, all data of each biomarker are shown for the 12 patients as a single study group.

The study medication was well tolerated and only 5 mild adverse events were possibly treatment-related. There were no clinically important changes in lung function or safety variables.

### Assay results

**Figure 1:**
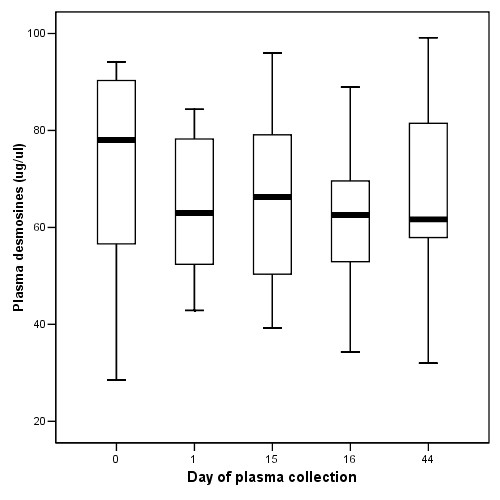
Plasma desmosines levels (median ± quartels presented as box together with minimal and maximal values) determined on samples collected on indicated days from the patients. Values of plasma desmosines in healthy individuals (n = 15) range between 40 and 60 μg/l, mean 43 ± 3 μg/l (mean ± SD, reference 16).

**Figure 2:**
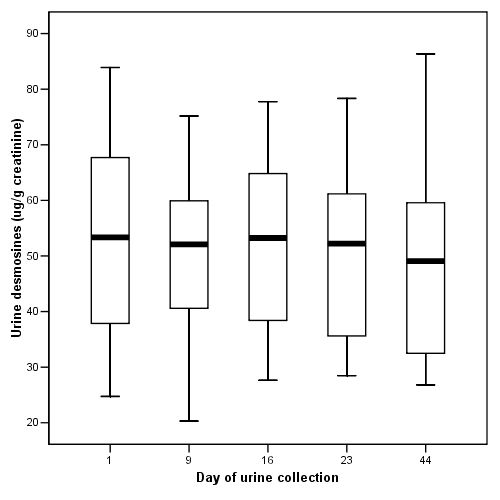
Urine desmosines levels (median ± quartels) determined on samples collected on indicated days from the study patients. Ten healthy individuals had mean values of 22.70 ± 1.66 μg/g creatinine (mean ± SD, reference 14).

**Figure 3:**
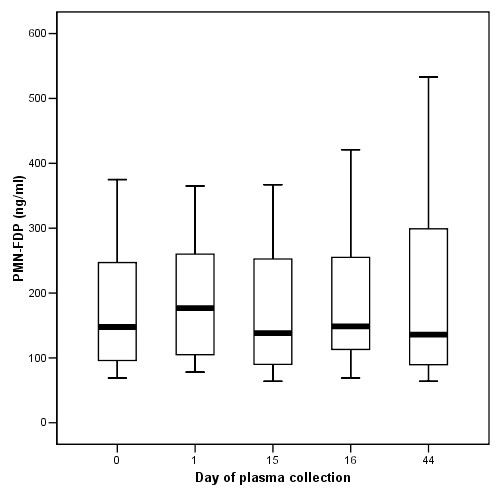
Plasma fibrinogen fragments generated by PMN (PMN-FDP) determined on samples collected on indicated days from the study patients (median ± quartels). Ten healthy individuals had mean values of 35 ± 12 ng/ml (mean ± SD, reference 11).

**Figure 4:**
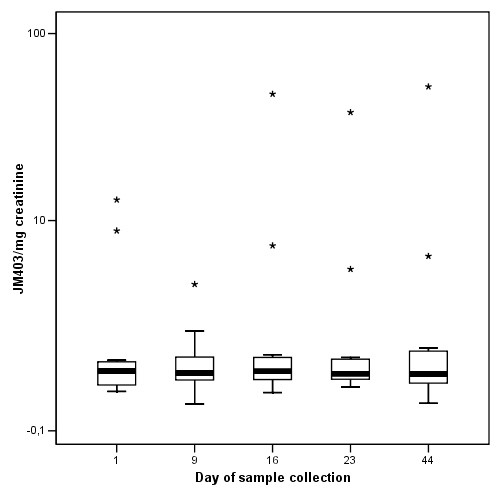
Urine JM403 values corrected for urine creatinine concentration on samples collected on indicated days from the study patients. Individual values are shown to appreciate the differences between patients. Values are expressed as Units/mg creatinine. One unit is defined as the amount of JM403 epitope present on 1 microgram kidney heparan sulfate. Sample on Day 9 of patient X is missing because he forgot to collect the sample. The top line of asterixes represents data from a patient that also had stable chronic pancreatitis, a condition not known to be associated with type Z alpha-1-antitrypsin deficiency. The other patient with normal values had no other known conditions and had a stable lung function for the past 15 years as measured in our clinic.

The mean values of desmosines and PMN-FDP of the 12 patients for each of the study days were all in the same range (Figure 1, 2, 3). Previously published values in healthy individuals are mentioned in the legend to the figures. Except for patient 4 and 10, JM403 values were within the range of values for patients with general COPD [7]. The JM 403 epitope in urine of patient 4 (FEV_1 _46% pred., Kco 40% pred.) and 10 (FEV_1 _30% pred., Kco 50% pred.) showed a markedly elevated value that was in the range of previously published values for healthy subjects (Figure 4) [7].

**Figure 5:**
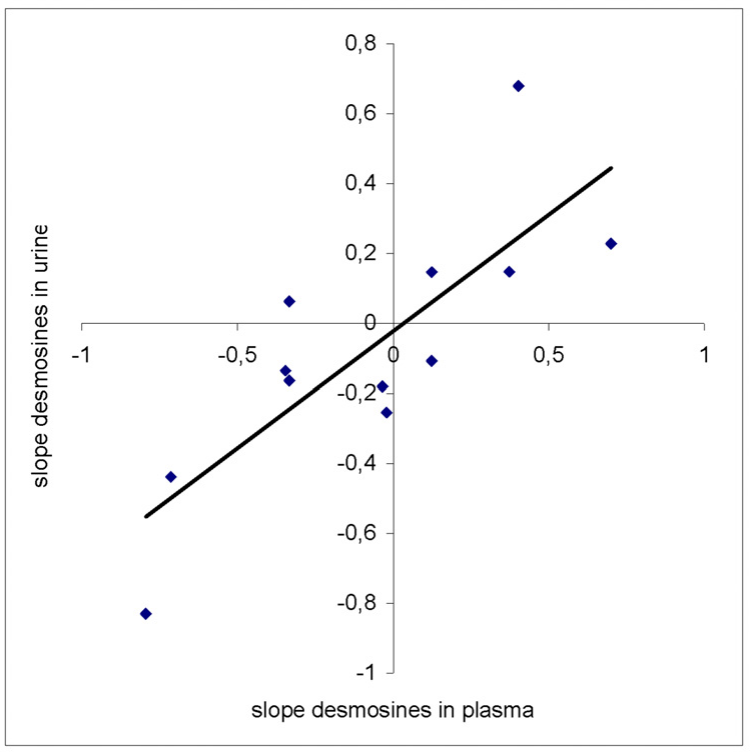
Change in plasma desmosines level and change in urine desmosines level during the 44 days of the study assessed for each of the 12 participating patients. Spearman's rank correlation coefficient between individual change in plasma and urine desmosines is 0.84 (P < 0.01).

Spearman's rank correlation coefficient between baseline lung function values of the patients and each of the baseline biomarkers is presented in Table 2 (see DOC AdditionalfileRespirResTable2). Spearman's correlation of change over time between plasma desmosines and urine desmosines in the 12 patients was 0.84 (P < 0.01) and is shown in Figure 5. No such significant correlation was present for the two other biomarkers.

The coefficient of variation (CV) calculated within and between patients is presented in Table 3 (see DOC AdditionalfileRespirResTable3). Mean coefficient of variation within patients (CVi) for plasma and urine desmosines was 18.7 to 13.5%, respectively. Mean CVi for fibrinogen fragments in plasma was 20.5% and for JM403 in urine was 27.8%.

## Discussion

The results of this 44-day follow up study showed for the first time the variability of biomarkers of matrix degradation within and between patients with Pi ZZ type of alpha-1-antitrypsin deficiency and emphysema. Furthermore, the present baseline results confirm values of similar patients in previously published cross-sectional studies where the outcome was compared with control subjects [7-9,11]. The patients in the present study were carefully controlled during the conduct of the study, including two overnight stays in the clinic and three visits in the out patient clinic, allowing optimal conditions for sample collection and preparation.

The assays were performed in three different laboratories, each with their own expertise built up in the past 5 years. For the JM403 epitope assay, urine samples needed specific pre-treatment [7]. Glycosaminoglycans were first isolated by an ion-exchange column and quantified. From the glycosaminoglycan eluate, a sample was used for the JM403 EIA. When values of our patients 4 and 10 with high JM403 levels are taken out, a mean of 0.7 ± 0.2 U/mg creatinine is highly comparable to the previously published value of 0.6 ± 0.1 U/mg creatinine, indicating stable assay performance of the EIA despite a complicated sample preparation. This is in contrast to the PMN-FDP assay [11]. We learned that cryo-precipitation of fibrinogen and large fibrinogen fragments in plasma samples requires careful sample handling, including snap freezing and rapid thawing to 37°C to prevent the formation of cryoprecipitates within the sample. In a previous report on this assay, our values for Pi ZZ patients were ten-fold lower, as these samples were thawed to and measured at room temperature [11]. To minimize solute losses in the system and to decrease the degree of variability of data, the determination of urinary desmosines was performed on urine samples not submitted to any pretreatment procedure other than filtering the sample. For all three assays, it is possible to measure values with an analytical inter-assay coefficient of variation (CVa) below 10%. This is well below 15% which is the value derived from the criterion that the CVa should be below half of the intra-individual coefficient of variation (CVi), with ranges between individuals reported in Table 3. Therefore, all three assays have acceptable variability and can be used for studies that aim to evaluate the effect of drugs on the level of these biomarkers [15].

The key question about all three of our assays is whether they only reflect matrix degradation present in lung interstitium or whether significant other parts in the body also contribute to elevated plasma levels of the biomarkers. Recent data showed abnormal high levels of desmosines in plasma and urine of patients affected by *Pseudoxanthoma elasticum*, an inherited disorder of connective tissue characterized by severe alterations of its components [16]. As COPD/emphysema is now recognised not only as a pulmonary condition, but also as a systemic disorder, including skeletal muscle wasting and skin atrophy, it is possible that other tissues in the body also contributed to the levels of our biomarkers due to the same processes. Furthermore, almost all patients with COPD/emphysema have a history of cigarette smoking that may also result in vascular atherosclerosis, a condition that is able to affect the levels of our biomarkers. To our knowledge, no lung specific protein, sensitive to proteolytic cleavage, has been identified yet to serve as a useful substrate for proteolysis to act as a footprint of inflammation-induced matrix destruction specific for the lung. Therefore, the most appropriate way to study the contribution of inflammation-induced lung matrix degradation would be the use of specific protease inhibitors in patients and to measure their effects on levels of our biomarkers. So far, urinary desmosines only has been used to monitor the efficacy of the AAT replacement therapy in short-term, open label trials in AATD subjects [17,18]. The latter study showed a small, but not significant, decrease in desmosines excretion after 24 weeks of replacement therapy, which suggests that reversal of chronic proteolysis in AATD cannot be achieved quickly. Just like in the above-mentioned studies, also our study was of short-term duration and no change in spirometry or CO diffusion capacity is to be expected. Therefore, a definitive answer to the question whether the increased urinary excretion in AATD-associated COPD is mostly due to accelerated proteolysis within the lung would require a more extended period of replacement therapy (probably more than one year) or a higher dose than currently used. Apart from above mentioned manuscripts, all the other reports dealing with matrix degradation biomarkers in COPD have been designed in a cross-sectional fashion [8,9,19].

A unique feature of the present study is that three different laboratories have united to measure three different biomarkers of matrix degradation in two different biological fluids being serially collected from a well-defined study population. Such collaboration arose from the Alpha_1 _International Registry (AIR), a consortium of scientists with special interest in AATD [20]. In this study, urinary and plasma desmosines have been detected simultaneously for the first time in these patients. Plasma normally contains peptides derived either from tropoelastin or from degraded crosslinked mature elastin. It has been reported that these circulating peptides have a wide variety of sizes, peaking at 70 kD, but with a significant proportion of peptides with lower molecular weight. The latter are expected to more easily filter into urine. Chromatographic separation of urine elastin peptides in humans has detected material over a wide range of MW, from < 5 kD to > 50 kD, peaking at 10–50 kD [21]. Detection of desmosines allows discrimination, among elastin peptides, of those derived from the breakdown of mature elastin from those derived from nascent elastin. The good correlation between urinary and plasma desmosines here shown is consistent with the correlation between urinary and plasma elastin peptides previously reported, suggesting that plasma and urine provide generally comparable estimates of elastin turnover, at least in a short-term [21]. The high cross-sectional correlation between baseline CO diffusion capacity and both urine and plasma desmosines and the absence of such correlation with either PMN-FDP or JM403 raises the question as to whether one is better than the other. Differential changes in any of these biomarkers during acute exacerbations or AAT supplementation therapy may answer this question. In addition, all three parameters do not seem to correlate with each other. This may in part be explained by a difference in clearance from the body. The half-life of desmosines-containing elastin fragments in mice is about 2 h [22], about 5 days for PMN-FDP in humans [23] and is unknown for JM403.

## Conclusion

We have shown, compared to previously reported healthy individuals, significantly altered levels of three different surrogate markers representing footprints of matrix degradation simultaneously present in patients with type Z alpha-1-antitrypsin deficiency and clinically significant emphysema. In addition, acceptable variability in our study parameters occurred. The footprints are recommended for use in an evaluation of biochemical efficacy of alpha-1-antitrypsin augmentation therapy in Pi Z subjects before initiation of studies involving functional tests of the lung and CT scan lung density assessment [24].

## Abbreviations

AAT, alpha-1-antitrypsin

AATD, alpha-1-antitrypsin deficiency

CE-LIF, capillary electrophoresis and laser-induced fluorescence detection

COPD, chronic obstructive pulmonary disease

FITC, fluoresceine isothiocyanate

HA, hyaluronic acid

EIA, inhibition enzyme immunoassay

Kco, CO diffusion capacity per volume unit of alveolar ventilation

PMN-FDP, neutrophil elastase-mediated degradation

PMNs, polymorphonuclear leukocytes

## Competing interests

Financial support: The study was conducted as a phase I study in collaboration with CoTherix, Belmont, CA, USA who financially supported the study.

All authors declared no conflict of interest with the content of the manuscript.

## Authors' contributions

All authors contributed to the content of the manuscript. The desmosines were measured by LA, CZ and PI. WN was responsible for the PMN-FDP assay. TK and EV performed the JM403 assay and BV performed the statistical analysis. JS and ML were responsible for the clinical work and JS wrote the manuscript. All authors have read the text and contributed corrections.
