# Correction: Short-term variability of biomarkers of proteinase activity in patients with emphysema associated with type Z alpha-1-antitrypsin deficiency

**DOI:** 10.1186/1465-9921-7-20

**Published:** 2006-02-03

**Authors:** Jan Stolk, Barbara Veldhuisen, Laura Annovazzi, Chiara Zanone, Elly M Versteeg, Toine H van Kuppevelt, Jo HM Berden, Willem Nieuwenhuizen, Paolo Iadarola, Maurizio Luisetti

**Affiliations:** 1 Department of Pulmonology, Leiden University Medical Center, Leiden, The Netherlands; 2 Department of Medical Statistics, Leiden University Medical Center, Leiden, The Netherlands; 3 Laboratory of Capillary Electrophoresis, Department of Biochemistry, University of Pavia, Italy; 4 Laboratory of Biochemistry and Genetics, Department of Respiratory Disease, IRCCS San Matteo Hospital, Pavia, Italy; 5 Department of Biochemistry, 194, University Medical Center, NCMLS Nijmegen, The Netherlands; 6 Department of Nephrology, Radboud University Nijmegen Medical Center, Nijmegen, The Netherlands; 7 TNO-Prevention and Health, Gaubius Laboratory, Leiden, The Netherlands

## Abstract

After the publication of this work [1], we became aware of the fact that one author was missing on the author list. Dr Jo.H.M. Berden contributed the JM403 antibody and advised on the methodology for the ELISA for JM403. He also contributed to the text of the manuscript.

Apart from this correction concerning the authorship, the methods and interpretation of the data, the results reported in our publication and the conclusions are absolutely correct.

We apologize for the inconvenience that this inaccuracy may have caused.
